# Antibiotics in Animal Feed and Spread of Resistant *Campylobacter* from Poultry to Humans

**DOI:** 10.3201/eid1006.040403

**Published:** 2004-06

**Authors:** Nicole M. Iovine, Martin J. Blaser

**Affiliations:** * New York University School of Medicine, New York, New York, USA;; † New York Harbor Department of Veterans Affairs Medical Center, New York, New York, USA

**Keywords:** antibiotics, animal feed, resistant campylobacter, poultry, humans

Contamination of food with potentially dangerous human pathogens has been recognized since the time of Pasteur (*1*) and is well-documented in the modern era (*2*), but the development of antimicrobial agents has helped limit the consequences of such infections. Concomitantly, the widespread use of antimicrobial agents has also led to the emergence of antimicrobial drug–resistant organisms (*3**,**4*). Gupta et al. demonstrate the increasing prevalence in the United States of ciprofloxacin-resistant *Campylobacter* species isolated from humans and poultry from 1990 to 1997, and their studies implicate the prophylactic treatment of poultry with fluoroquinolones in this emerging problem (*5*). Their report indicates that the source of fluoroquinolone-resistant *Campylobacter* infections was consuming poultry colonized with resistant strains (Figure), rather than selection for *Campylobacter* resistance in the human gut after clinical fluoroquinolone use to treat the diarrheal illness (*5*). This work provides further evidence that fluoroquinolone use in poultry promotes the emergence of resistant *Campylobacter* strains that subsequently infect humans (*6*). That persons infected with these fluoroquinolone-resistant strains had 3 additional days of illness and were more likely to be hospitalized demonstrates the harm caused by such resistant stains (*5*).

**Figure:**
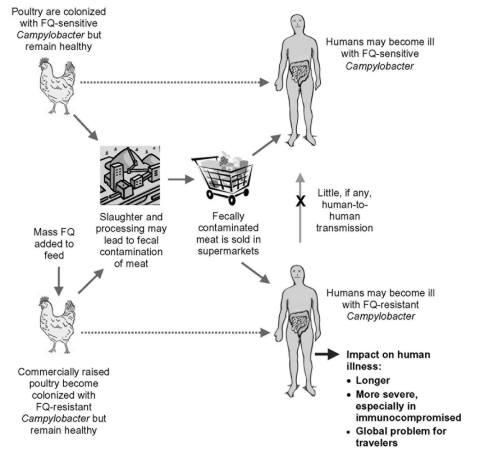
Acquisition of fluoroquinolone (FQ)-resistant *Campylobacter* from poultry.

Since campylobacters are normal enteric flora in many avian species, poultry represents a model system to test the hypothesis that prophylactic and growth-promoting use of antimicrobial agents in food animals selects for the emergence of antimicrobial drug–resistant organisms. In one study, chickens that were naturally colonized with fluoroquinolone-susceptible *Campylobacter* strains began to excrete resistant strains after 2 days of doses of enrofloxacin (*7*), which is commonly used for prophylaxis in the poultry industry. A single point mutation in *gyrA* encoding the bacterial DNA gyrase was sufficient to confer high-level resistance (*7**,**8*). This small genetic change apparently has a low "fitness cost" to the organism, as evidenced by fluoroquinolone-resistant strains' rapidly replacing susceptible *Campylobacter* in treated chickens (*7*). Developing an animal reservoir of fluoroquinolone-resistant *Campylobacter* has been the major factor behind transmission of quinolone resistance to humans (*8**,**9*).

In contrast, among poultry treated therapeutically with enrofloxacin, no resistance was observed in the 13 *C. jejuni* isolates tested (*9*). Similarly, after the prophylactic and growth-promoting uses of macrolides in swine were banned in Denmark, the prevalence of macrolide-resistant *C. coli* declined (*10*). Thus, the major determinant of developing resistance appears to be use of subtherapeutic antimicrobial doses. The antimicrobial drug ban in Denmark did not decrease the amount of meat produced by the poultry and pig production industries, which removed a major concern (*10*). Evidence suggests that restricting fluoroquinolone use to therapeutic indications only in food animals could decrease rates of fluoroquinolone-resistant *Campylobacter*, and the Danish experience with macrolide restriction proves that such limitations need not harm the husbandry of food animals.

The increased likelihood of foreign travel in persons infected with ciprofloxacin-resistant strains (*5*) illustrates the global threat posed by resistant strains. Appreciating such realities favors concerted efforts to limit use of fluoroquinolones (and other antimicrobial drugs) to therapy only in food animals. This view was supported by a recent (March 2004) landmark decision by Federal Drug Administration Administrative Law Judge Daniel J. Davidson, withdrawing approval for the new animal drug application to use enrofloxacin for prophylaxis or growth-promotion in poultry (*11*). This decision was the first occasion that a previously approved antimicrobial agent was removed from the U.S. veterinary market because of concerns about antimicrobial drug resistance. With this decision as precedent, we should follow the examples set in Europe and ban use of all antimicrobial agents in food animals, except when necessary for therapy of ill animals.
